# Innate and adaptive immunity to SARS-CoV-2 and predisposing factors

**DOI:** 10.3389/fimmu.2023.1159326

**Published:** 2023-05-09

**Authors:** Jiaying Shen, Junyan Fan, Yue Zhao, Doming Jiang, Zheyun Niu, Zihan Zhang, Guangwen Cao

**Affiliations:** ^1^ Tongji University School of Medicine, Tongji University, Shanghai, China; ^2^ Department of Epidemiology, Shanghai Key Laboratory of Medical Bioprotection, Key Laboratory of Biological Defense, Ministry of Education, Second Military Medical University, Shanghai, China

**Keywords:** COVID-19, SARS-CoV-2, immune response, viral immune evasion, susceptibility

## Abstract

The coronavirus disease 2019 (COVID-19) pandemic, caused by severe acute respiratory syndrome coronavirus (SARS-CoV-2), has affected all countries worldwide. Although some symptoms are relatively mild, others are still associated with severe and even fatal clinical outcomes. Innate and adaptive immunity are important for the control of SARS-CoV-2 infections, whereas a comprehensive characterization of the innate and adaptive immune response to COVID-19 is still lacking and the mechanisms underlying immune pathogenesis and host predisposing factors are still a matter of scientific debate. Here, the specific functions and kinetics of innate and adaptive immunity involved in SARS-CoV-2 recognition and resultant pathogenesis are discussed, as well as their immune memory for vaccinations, viral-mediated immune evasion, and the current and future immunotherapeutic agents. We also highlight host factors that contribute to infection, which may deepen the understanding of viral pathogenesis and help identify targeted therapies that attenuate severe disease and infection.

## Introduction

Coronavirus disease 2019 (COVID-19), caused by severe acute respiratory syndrome coronavirus 2 (SARS-CoV-2), has been declared as a global health emergency, which is characterized by fever, respiratory illness, and pneumonia and other symptoms. According to the Johns Hopkins Coronavirus Resource Center (1), there have been more than 650 million confirmed positive cases worldwide, and 6 million deaths worldwide by December 2022. SARS-CoV-2 has a wide range of hosts and constantly increasing ability to transmit and immune escape, thereby probably coexisting with humans for a long time. So far, further studies are needed to elucidate mechanisms by which host immunity against SARS-CoV-2. Which and to what extent factors may account for the predispositions of individuals to contract infection remains unclear. Therefore, it is necessary for us to have an updated understanding of the interaction between SARS-CoV-2 and host immunity and the susceptible factors to viral infection.

SARS-CoV-2 is an enveloped, positive-sense, single-stranded RNA virus with approximately 30kb in size (2). As a member of the coronavirus family, SARS-CoV-2 has four structural proteins, including spike (S), envelope (E), membrane (M), and nucleocapsid (N) and non-structural proteins (NSP-1-16) (3). The spike protein is composed of two functional subunits, including the S1 and S2 unit. The function of S1 is to bind the receptor on the host cell, while S2 is responsible for fusing the membranes of the virus and the host cell. Additionally, angiotensin-converting enzyme 2 (ACE2) is its major cellular receptor (4) and the transmembrane protease serine protease-2 (TMPRSS-2) and cathepsin L are used for S protein priming (5). The expression of ACE2 and TMPRSS2 is heterogeneously expressed in different organs (6) and tissues with more than 1% ACE2 expression proportion are considered at higher risk of infection, including lower respiratory tract (2%), lung (> 1%), heart (> 7.5%), ileum (3%), esophagus (> 1%), kidney (4%), and bladder (2.4%) (7). ACE2 also regulate coagulation and inflammation during viral infection in the renin angiotensin system (RAS) (8). TMPRSS2 expression corresponds to ACE2 expression in many tissues, including kidney, liver, testis, small intestine, and lung (9–12).

Like SARS-CoV and MERS-CoV, SARS-CoV-2 has been investigated to clarify the characterization of innate and adaptive immune response (13, 14). Innate immune cells can recognize pathogen-associated molecular patterns (PAMPs) through cytosolic pattern recognition receptors (PRRs) to limit SARS-CoV-2 replication and promote viral clearance (15). Interleukin (IL)-6 has been considered as a potential pathogenic factor in the initiation of acute respiratory distress syndrome (ARDS) (16). Adaptive immunity involves the co-ordination of T cells and B cells to control SARS-CoV-2 (17). Adaptive immune responses to SARS-CoV-2 occur within the first 7-10 days post-infection (17). However, the nature of the B and T cell immune events and their long immunity remain unclear, which is an important issue for vaccine development. On the other hand, an over-activated or aberrant immune response can lead to immunopathology and more severe symptoms, such as tissue damage, acute respiratory distress syndrome, thromboembolic complications, cardiac injury and/or cytokine storm (15). Therefore, understanding the underlying immune response is important for risk stratification and clinical triage.

Currently, Omicron variants are becoming the dominant strain of COVID-19. They have evolved into many sublines, such as BA.1, BA.2, BA.2.12.1, BA.4, and BA.5. Unlike other variants of concern (VOCs), it is characterized by a high proportion of asymptomatic cases, and a low mortality rate (18). Nearly 7.9%-61.0% of infected individuals remained asymptomatic and some showed no symptoms in the early stage (19). A number of factors have been identified to have a risk or protective impact on developing severe clinical outcomes (20). However, studies on human factors associated with the susceptibility to COVID-19 are relatively rare. As the lethality and virulence of SARS-CoV-2 continue to decline and more asymptomatic cases occur, the need to identify its predisposing factors to prevent infection or reinfection becomes more urgent.

**Figure 1:**
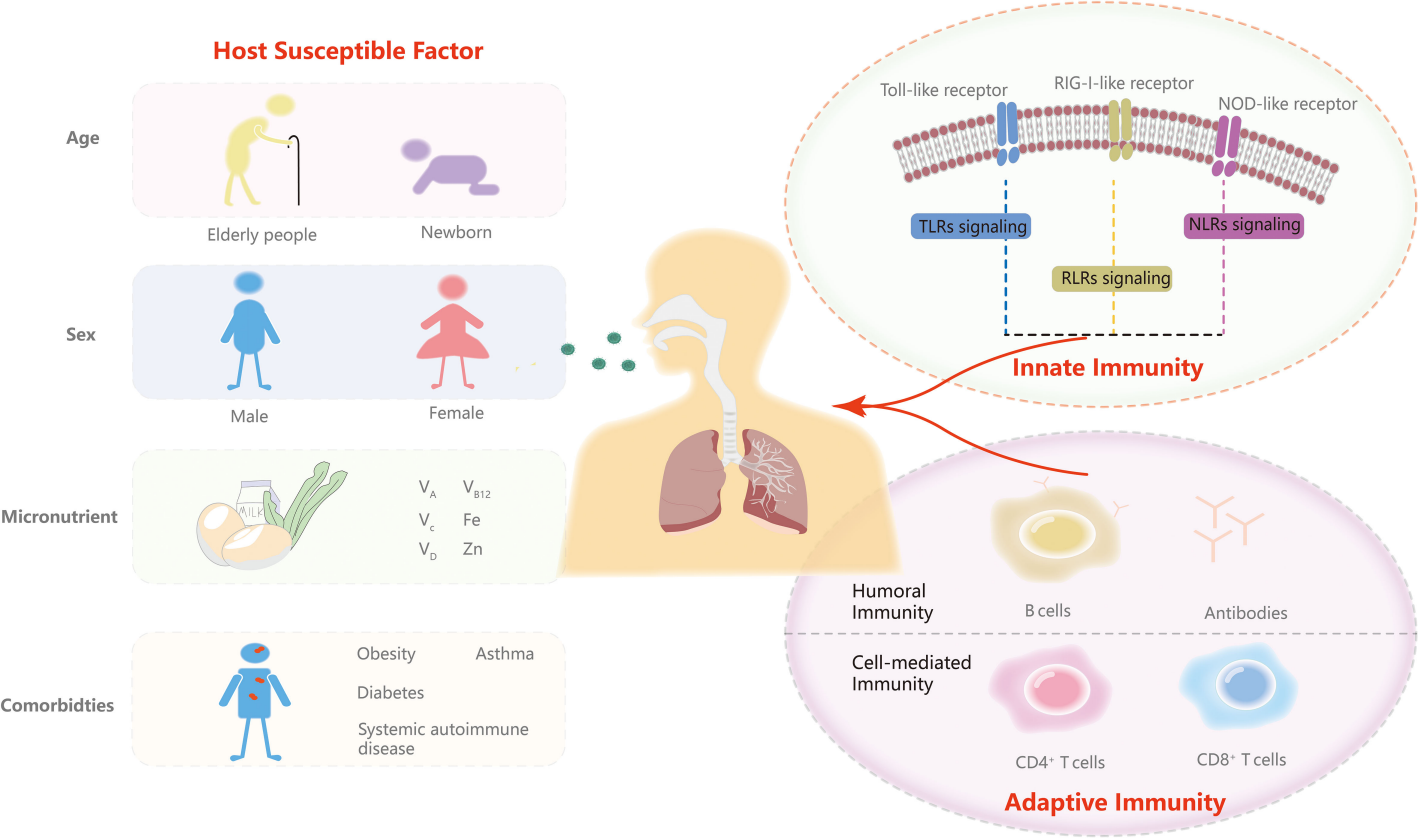
Innate and adaptive immunity and predisposing factors. In this review, we discuss the three major PRRs signaling response including TLRs signaling, RLR signaling and NLR signaling of innate immunity and T and B cells of adaptive immunity. We then highlight the role of age, sex, micronutrients and comorbidities in the predispositions to SARS-CoV-2.

In this article, we update the contributions of major pattern recognition receptors (PRRs) in innate immunity and T and B cells in adaptive immunity and vaccination. An overview of immunotherapy for COVID-19 is presented in this study. We also highlight mechanisms by which viruses evade anti-COVID-19 immunity. Moreover, we discuss host susceptible factors about how they influence infection. Continuing to improve our understanding of the immune system and host susceptibility is critical to achieving translation from molecular mechanisms and therapy to prevention strategies in the post-COVID-19 era (**Figure 1**).

## Innate immune system

**Figure 2:**
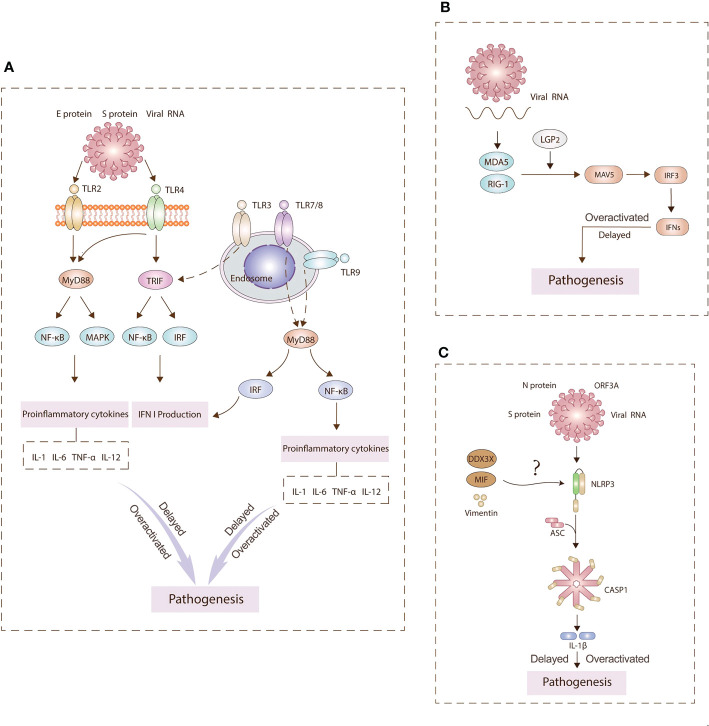
Main pathogenesis mechanisms underlying TLRs **(A)**, RLRs **(B)**, NLRs **(C)** signaling. PRRs on the cell surface and endosomal membranes and in the cytosol detect SARS-CoV-2 antigens to activate innate immunity signaling pathways. TLR2 and TLR4 can signal through MyD88 to activate NF-κB and MAPK signaling pathways to generate proinflammatory cytokines. TLR4 and TLR3 can signal TRIF to activate IRF3 and induce type I IFN expression. TLR7/8 and TLR9 can signal through MyD88 to activate NF-kB and IRF to induce IFN I production and inflammasomes. Hyperinflammation leads to pathogenesis. RLRs sense viral RNA and engages IRF3 to produce IFNs. The NLRP3 inflammasome is assembled following sensing of spike (S) and nucleocapsid (N) proteins, viral RNA, and open reading frame 3A (ORF3A). This assembly leads to the production of interleukin (IL)‐1β. Overall, the delay or overactivation of these three signaling can drive COVID‐19 pathology.

During the entry of SARS-CoV-2, viral binding to the ACE2 receptor induces conformational changes in the S1 subunit (21). S2’ cleavage then occurs in the presence of cellular proteases such as TMPRSS2 or cathepsin L (22). This series of viral infection is detected by a variety of host PRRs (15). The three major PRR families include Toll-like receptors (TLRs), retinoic acid-inducible gene I (RIG-I)-like receptors (RLRs), and nucleotide-binding oligomerization domain (NOD)-like receptors (NLRs) (23). The aberrant signaling pathways through these receptors over-activate inflammatory cytokines and chemokines (24) (**Figure 2**). A better understanding of pathophysiological mechanisms involved in innate immunity is a prerequisite for developing curative and preventive strategies against COVID-19.

### TLRs and SARS-CoV-2

TLRs are the “gatekeepers” of the human immune system to protect the host from invading pathogens. TLRs have ten family members. Some are in the cell membrane, while the others are situated in endosomes. Since SARS-CoV-2 is a single-stranded RNA virus, at least six TLRs have been implicated in viral recognition, namely TLR2, TLR3, TLR4, TLR7, TLR8 and TLR9. TLR2 and TLR4 can recognize viral structural and nonstructural proteins outside the cell (25). SARS-CoV-2 spike protein S1 subunit has been found to activate TLR4 signaling to induce pro-inflammatory responses in human macrophages (26). TLR2 can sense the SARS-CoV-2 envelope protein to produce inflammatory cytokines (27). TLR3 can identify double-stranded RNA during viral replication. After SARS-CoV-2 is engulfed by macrophages, the genomic RNAs released from the virions are recognized by TLR7/TLR8, thereby stimulating downstream signaling pathways. TLR7 is mainly involved in the control of innate immunity during pulmonary SARS-CoV-2 infection, activating the NF-kB transduction and leading to pro-inflammatory cytokine secretion (28, 29). In contrast, relevant research on TLR9 is still rare. When SARS-CoV-2 infects endothelial cells, mitochondrial dysfunction elevates mtDNA levels and induces TLR9 signaling (30). Myeloid differentiation primary response 88 (MyD88) and TIR domain-containing adapter-inducing interferon-β (TRIF) are two major pathways for the transduction of TLR’s signals. MyD88-dependent pathway culminates in the activation of both nuclear factor kappa-B (NF-κB) and mitogen-activated protein kinase (MAPK) to stimulate pro-inflammatory cytokines, while the TRIF-independent pathway culminates in the activation of NF-κB and interferon regulatory factor (IRF) to produce type I IFN and pro-inflammatory cytokines, such as interleukin-1 (IL-1), IL-6, tumor necrosis factor‐α (TNF-α), and IL-12. On the other hand, TLRs can also harm the host by causing persistent inflammation and tissue destruction. The interaction of TLRs with viral particles leads to the production of 1L-1β which is positively associated with the immunopathological consequences, including death. TLR4 may contribute significantly to the pathogenesis of SARS-CoV-2 by inducing aberrant hyperinflammation (31, 32).

### RLRs and SARS-CoV-2

RLRs encompass three homologous members, including RIG-I (or DEAD box polypeptide 58, DDX58), melanoma differentiation-associated gene 5 (MDA5), and laboratory of genetics and physiology 2 (LGP2) (33). During SARS-CoV-2 infection, RIG-1 and MDA5 are mainly involved in identifying viral RNA and inhibiting viral replication by recognizing viral intermediate dsRNA. Usually, activated RLRs are interacted with mitochondrial antiviral-signaling protein (MAVS) to regulate IFN I and III pathways (34). The activity of subsequent ISGs including LY6E, AXIN2, CH25H, EPST1I, GBP5, IFIH1, IFITM2 and IFITM3 has been found to inhibit replication and entry of SARS-CoV-2 (35). However, SARS-CoV-2 can inhibit RLR signaling in a deubiquitination-dependent and deubiquitination-independent manner through its papain-like protease to interfere immune response (36). Noticeably, children have higher basal expression of RIG-1 and MDA5 in upper airway epithelial cells (37), resulting in a stronger and earlier initial antiviral response to SARS-CoV-2 than adults.

On the other hand, the conclusions of current studies are inconsistent in the role of RLRs signaling in the viral recognition and activity. RIG-1 is found able to recognize the 3’ untranslated region of the SARS-CoV-2 RNA genome through the helicase domains, rather than the canonical C-terminal domain of RIG-I, and directly abrogate viral RNA-dependent RNA polymerase mediation of the first step of replication in a type I/II interferon (IFN)-independent way (38). In contrast, another study has concluded that RIG-1 and MDA5 can initiate an antiviral state by increasing the expression of cytokines and interferon (IFN)-stimulated genes (ISGs), such as CCL5 and IFN-β (39). However, the IFN-β expression is not found to be affected in another study when RIG-I is silenced in Calu-3 cells. MDA5 and LGP2 are found to primarily regulate IFN induction in response to viral infection by screening 16 related sensors (34).

### NLRs and SARS-CoV-2

There are four main types of NLRs: NLRP1, NLRP3, NLRC4 and AIM2. The present studies are organized around their roles in SARS-CoV-2 infection. The elevated NLRC4 in zebrafish could promote the antiviral response and regulate the MDA5-MAVS and TRAF3-MAVS complexes, thereby modulating the transcription of type I IFNs and interferon-stimulated genes (ISGs) (40). In contrast, when blood monocytes are infected, NLRP3 and AIM2 should be activated, leading to pyroptosis and cytokine induction (41). Highly expressed NLRP1 often leads to more complications of systemic cardiovascular diseases compared with MERS and SARS (42). Among them, NLRP3 is the best studied inflammasome, which consists of a leucine-rich repeat (LRR), a central nucleotide-binding domain (NACHT), a pyrin domain (PYD) and a caspase recruitment domain (CARD). Normally, the formation of the NLRP3 inflammasome requires two steps. The first step is the induction of NF-κB, activated by various PAMPs and DAMPs, resulting in the elevated pro-IL-1β, pro-IL-18 and NLRP3. The subsequent signals are derived from various inducers, among which potassium (K+) efflux is the necessary trigger for NLRP3 inflammasome assembly (43). Once activated by these signals, NLRP3 is oligomerized and assembled with apoptosis-associated speck-like protein containing a CARD (PYCARD, also known as ASC) and pro-caspase-1 (44).

Several SARS-CoV PAMPs derived from ORF3a, ORF8b, E protein and viral RNA, can activate the NLRP3 inflammasome (45). In human very small embryonic-like stem cells (VSELs) and hematopoietic stem cells (HSCs), NLRP3 inflammasome assembly can also be initiated by the interaction of ACE2 with S protein and N protein of SARS-CoV-2. DEAD-box helicase 3X (DDX3X), vimentin, and macrophage migration inhibitory factor (MIF) play a significant role in activating NLRP3 formation during SARS-CoV-2 infection (44). A co-immunoprecipitation analysis suggested that DDX3X might integrate the central NACHT domain of NLRP3 to promote its assembly, although more direct evidence is required. Vimentin has been suggested to bind the LRR domain of NLRP3 to interact with signaling molecules. Less IL-1β and IL-18 are secreted in the MIF -/- mice and cells, but the MIF inhibitors were found in some studies to have no effect on NLRP3 production (46).

However, uncontrolled NLRP3 inflammasome can trigger excessive IL-1β and other inflammatory cytokines, leading to the cell death by pyroptosis (47, 48). Excessive mature IL-1β can stimulate systemic inflammatory responses, resulting in the release of large amounts of cytokines, including IL-6, tumor necrosis factor-α (TNFα), interferon (IFN)-α, IFN-β. NLRP3 inflammasome genetic variants are associated with severe COVID-19-related disease (49). NLRP3 inflammasome aggregates pulmonary injury in ARDS-related patients. This hyper-expression of NLRP3 inflammasome may be due to the impaired mitochondrial function and over-generation of reactive oxygen species (mtROS), especially in elderly population, leading to the over-activation of classical activated macrophage (M1) (50). In SARS-CoV-2 infected hACE2 mouse models, those orally administered the NLRP3 inhibitor had significantly decreased microglial inflammasome activation and higher survival rate, compared with those in untreated groups (51). Therefore, blocking NLRP3 pathway is considered feasible in drug therapy to attenuate cytokine release in patients (52). Probiotics can also be suppressed the NLRP3 inflammasome without affecting normal immune function (53).

## Adaptive immune system

**Figure 3:**
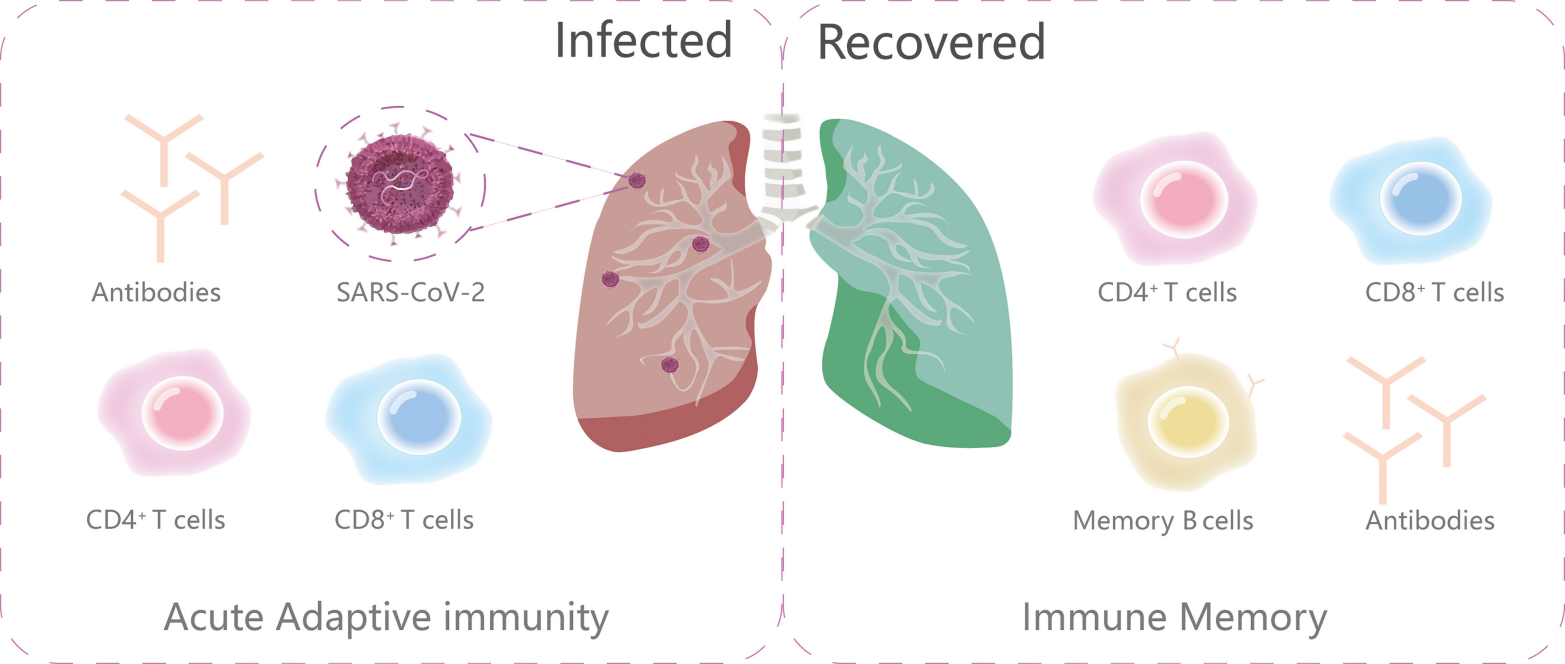
The primary components of adaptive immunity during and after infection. The ongoing adaptive immunity to viral infections consists of three main parts: virus-specific CD4+T cells, CD8+T cells and antibodies. After infection, the immune memory is subsequently made up of memory B cells, antibodies, virus-specific CD4+T cells and CD8+T cells.

Abundant evidence reveals that B cells (antibody producing cells), CD4^+^ T cells (helper T cells), and CD8^+^T cells (killer cells) all contribute to the control of SARS-CoV-2 (54, 55) (**Figure 3**). As the intricate interplay between the host background and key molecules in the adaptive immunity can influence the magnitude, longevity and the protective and/or pathological disposition of host immune response, we discuss the T and B cell immune events and span of long-lasting immunity after SARS-CoV-2 infection here.

### Response of B cells to SARS-CoV-2 infection

Humoral immune responses to SARS-CoV-2 appears to be induced at the onset of COVID-19. Formulation of immunologic memory requires two steps. The initial exposure to a viral pathogen elicits a plasmablast response to induce low-affinity antigen-specific B cells (56). Then, the CD4+ follicular helper T (Tfh) cells and B cells in the secondary lymphoid tissues facilitate antibody affinity maturation and isotype switching in a complex manner that generates long-term immune protection (57). It is noteworthy that the production of neutralizing antibodies by B cells is relatively fast and easy, as these antibodies are present in both heavy and light chain forms and almost no somatic hypermutation occurs. Unlike previous viral infections, including Dengue and Zika virus, serum IgG responses to SARS-CoV-2 occur at approximately the same time as serum IgM and IgA, usually within 7-10 days following the infection (55). IgG antibodies to the RBD domain are positively associated with anti-S neutralizing antibody titers, which have demonstrated little to no decline over 75 days after symptom onset (58). IgG antibody titers are relatively robust for at least 5 months after infection (59), which is associated with a significantly reduced risk of reinfection (60). IgG antibodies are also found to decline from 5–7 to 34–42 weeks, while 16.7% of patients are seronegative for IgM antibodies after 8-11 weeks (61). Circulating antibody levels may be related to many elements, such as the decline in response, the magnitude of the peak response, the subtypes of antibodies, and the relative contribution of short-lived and long-lived plasma cells (13, 55). Besides, patients with more severe symptoms often have higher peak neutralizing antibody titers (62), as high levels of viral antigen in patients tend to induce higher antibody titers. However, antibody titers in some patients are incredibly low, implying that there may be other ways for the virus to respond in adaptive immunity. Another study has found that symptomatic patients tend to become negative for antibody earlier than asymptomatic patients (63). More studies are needed to characterize the duration of antibody response between symptomatic and asymptomatic patients for the long-lasting immune response.

Given the high homology of SARS-CoV-2 with other human coronaviruses (HCoVs), it is hypothesized that these viruses may induce cross-reactive immunity, including HCOV-HKU1, HCOV-OC43, HCOV-NL63, HCOV-299E, Middle East respiratory syndrome coronavirus (MERS-CoV), and SARS-CoV. This pre-existing immunity may affect COVID-19 disease outcome (64). Understanding this immune process can allow us to identify conserved immune epitopes and facilitate vaccine development against SARS-CoV-2 and even future novel pandemic coronaviruses (65). Cross-reactive antibodies are evident in samples from SARS-CoV-2-unexposed individuals, including cross-reactivity of SARS-CoV-2 IgG antibodies with all four spike proteins of SARS-CoV, MERS-CoV, HCoV-OC43, and HCoV-HKU1 (66). Similarly, an analysis of 350 SARS-CoV-2-uninfected individuals displayed that the neutralizing antibody from uninfected donors might target the S2 subunit, rather than the RBD and the S1 subunit (67). Memory B cell populations of HCoVs have also been reported to possess cross-reactive immunity. Nine monoclonal antibodies isolated from the memory B repertoire of SARS-CoV samples showed potent cross-neutralization to SARS-CoV-2, eight of which targeted the domain that binds to ACE2 (68). Another cross-reactive neutralizing antibody specific to the S2 subunit of the S protein has also been identified from the pre-pandemic period (69). However, sera from recovered SARS patients are found to have the modest neutralizing activity against SARS-CoV-2 (70). Therefore, the cross-reactivity of antibodies from past coronaviruses (CoVs) to SARS-CoV-2 warrants further study.

### Response of T cells to SARS-CoV-2 infection

SARS-CoV-2 triggers the stimulation and recruitment of CD4^+^ and CD8^+^ T-cells which can control intracellular pathogens and eliminate virions. T cells mainly target S protein, M protein, N protein and non-structural protein (including NSP3 and NSP4), and ORF3a protein (71). SARS-CoV-2-specific CD4^+^ T cells usually differentiate into a series of helper cells and effector cells, including type1 helper T cells (Th1), Th17, follicular helper T cells (Tfh), regulatory T cells (Treg), and CD4^+^ cytotoxic T cells (CD4^+^ CTL) (72), which can be detected 2 - 4 days after the onset of the disease. Early cytotoxic CD8^+^T cells usually appear within 7 days and then kill infected cells. It is crucial to have a deeper understanding of their functions in the immune response to SARS-CoV-2, which will provide information for future studies of cellular immunity.

Delayed or overactivated T cell immune response can cause the severity of COVID-19. Deceased COVID-19 patients are manifested by impaired Tfh function and germinal center development, implying a significant role of Tfh cells in the recovery from COVID-19 (73). In macaques treated with a vaccine or natural virus, the absence of T cells delays virus clearance (74). CD4^+^PD-1CD57^+^ exhausted T cells are found in COVID-19 patients (75). On the other hand, the expression difference of CD4^+^T cells and CD8^+^T cells may be observed between severe and mild patients. The levels of IFN-γand TNF-αin CD4^+^T cells are lower in the severe group than in the mild group, where the levels of granzyme B and perforin in CD8^+^T cells are higher in the severe group than in the mild group (76), implying that SARS-CoV-2 infection may impair CD4^+^T cells and over-activate CD8^+^T cells in different COVID-19 status. However, CD4^+^T cells are observed to be high in the lungs of some severe COVID-19 patients, and CD4^+^ T cells expressing CD25 secrete the protease furin and facilitate the entry of SARS-CoV-2 (77). Overall, T cell immune response is found to be stronger in patients with more severe infection (78). In the last stages of severe COVID-19, T cells has a higher degree of proliferation, activation, and cytotoxicity (79). The overstimulation of combined CD8^+^ T, Th1, Th17 and NK cells also induce additional cytokines to target virus-infected cells, which lead to tissue damage (22).

There are six HCoVs that have relative amino acid conservation with SARS-CoV-2, including SARS-CoV-1, MERS-CoV, HCoV-OC43, HCoV-HKU1, HCoV-NL63, and HCoV-229E, and therefore their T cell epitopes may cause relative clinical protection from SARS-CoV-2. Most studies have found this cross-reactivity response (71, 80, 81), displaying that SARS-CoV-2-reactive T cells, which originate from previous exposure to the other coronaviruses have been found in unexposed individuals. Besides, a stronger cross-reactivity is positively associated with superior cellular immunity and better clinical outcomes (82). The contributions of these four coronaviruses to T-cell cross-reactivity remain undefined and the molecular mechanisms for cross-reactive recognition of SARS-CoV-2 is still unclear (83). Among HCoVs, SARS-CoV-1 and MERS-CoV are more homologous to SARS-CoV-2 but less prevalent, while the other four coronavirus diseases, HCoV-OC43, HCoV-HKU1, HCoV-NL63, and HCoV-229E, are less similar to SARS-CoV-2 but more widely spread (84). The phenomenon of this cross-reactivity has been suggested to result from exposure to these four more common human coronaviruses, as they circulate more widely in humans, which helps us better understand the clinical types and manifestations of COVID-19 (85, 86). Noticeably, children without previous SARS-CoV-2 infection mount higher levels of cross-reactive antibodies to subunit S2 of spike protein than adults (87), implying that children may have a stronger cross-reactivity response to promote viral clearance. Although the reason why the cross-reactivity is related to age remains unclear, these findings may help guide the design of pediatric vaccination regimens (88).

### Response of B and T cells following COVID-19 vaccination

The waning of humoral immunity over time suggests that mass vaccination may be a key strategy to control the COVID-19 epidemic. The current vaccines include Pfizer BNT162b2, Moderna mRNA-1273 and ChAdOx1 nCoV-19. After second dose of BNT162b2, the vaccine efficacy retains 95% at 7 days to 2 months, decreasing to 90% at 2-4 months and 84% at 4-6 months (89). The efficacy of the third dose of the BNT162b2 vaccine has been reported to be 95.3% (90). The two-dose regimen of Moderna mRNA-1273 is 94.1% effective in preventing symptomatic SARS-CoV-2 infection (91). After seven months, a neutralizing effect of Omicron has been detected in only 55% of participants (92). An efficacy of 93.2% against COVID-19 has been observed in people vaccinated with the booster after 5.3 months of follow-up (93). The effectiveness of ChAdOx1 nCoV-19 is about 72.8% in subjects aged 18-64 years and 82.5% in subjects aged 64 years and older (93). Specific-CD4^+^ cells and CD8^+^T cells are commonly used to evaluate the effectiveness of vaccines as a durable antibody response requires coordinated T and B lymphocyte interactions within lymphoid tissue germinal centers to produce long-lived plasma cells and switched memory B cells (55).

A vaccine can facilitate the immune response of B cells through the following pathways (94): (1) ongoing antibody somatic mutation; (2) clonal turnover of memory B cells; (3) development of monoclonal antibodies targeting RBD mutations. mRNA vaccines can trigger a stable class-switched memory B cell (MBC) response, which is significant in inducing a memory recall upon re-exposure to SARS-CoV-2. The MBC response can be further enhanced by administration of the second dose (95). A single dose of the BNT162b2 or the mRNA-1273 vaccine in seropositive patients induces the same IgG titers as seronegative individuals receiving two doses of vaccine (96). Subjects receiving three doses of an mRNA vaccine have a more effective memory B cell repertoire (97). Therefore, the booster may be required to prolong antibody response time (55). However, there is no increase in antibodies or the B cell memory response after the second dose in those previously infected individuals (98). On the other hand, there is concern that neutralizing antibodies (Nabs) to SARS-CoV-2 may decline over time and that some Nabs may paradoxically enhance SARS-CoV-2 infection by promoting syncytium formation (57). Vaccine recipients have been shown to recover fewer high affinity mature MBCs and to respond less efficiently to variants of concern (VOCs) than recovered patients (99). Therefore, more longitudinal studies are required to investigate the duration of vaccine-induced antibody titers.

The vaccine-induced immune response can induce relatively robust and durable T cell responses against the virus, manifested by activated CD4^+^ T cells and antigen-specific CD4^+^T cells (100). After booster vaccination, S1-specific T cell responses are generated and increase in one month, begin to decrease slightly after four months, but remain stable for seven months (101). The S-specific Tfh cells peak after the second dose and persist for at least six months (102). Vaccinated individuals have also been reported to develop higher levels of CD4^+^T cell activation than recovered COVID-19 patients (103). Spike-specific CD4^+^ and CD8^+^ T cells are elevated by 5.9 and 2.7 times, respectively, after the third dose (104). Notably, patients treated with CD20 B-cell-depleting therapy have a significantly reduced T cell immune response after the third booster, compared with healthy controls (105). Although the T cell response has been considered independent, there remains a gap in the knowledge of the association among the B cells, neutralizing antibodies, and T cells in the vaccine-induced immune response. A recent related study has found that previously infected people with a single dose of vaccination produce more specific memory B cells and distinct types of SARS-CoV-2 spike-specific CD4^+^T cells expressing IFN-γ and IL-10, compared with the uninfected individuals (106). It is hypothesized that T cell activation may be delayed and terminated in the absence of B cells.

## Immunotherapy against COVID-19

Isolation and close observation may be preferred treatment for asymptomatic SARS-CoV-2 carriers, as antiviral drugs are not found effective in improving viral clearance on asymptomatic infections (107), and even have side effects, such as liver impairment (108). For some severe patients, numerous immunotherapeutic interventions are under investigated to identify the most efficacious regimen (109).

In addition to antiviral drugs (remdesivir, molnupiravir, PF-07304814, ribavirin, favipravir, nafamostat, camostat, and aprotinin) (110–112), mAbs are therapeutic options, which can disrupt the interaction of the RBD of the S1 subunit in the Spike protein with ACE2 (113), resulting in reduced viral load and hospitalization rates. Typically, mAbs isolated from the B cells of recovered COVID-19 patients are potential therapeutic agents. For instance, S230 (114), CR3014 (115), and 80R (116) are three SARS-CoV neutralizing monoclonal antibodies that can also bind to the SARS-CoV-2 RBD. Currently, the FDA has granted emergency approval for the mAb combinations bamlanivimab with etesevimab and casirivimab with imdevimab (117). Treatment with the subcutaneous casirivimab and imdevimab antibody combination significantly reduces the incidence of symptomatic infection in asymptomatic respondents (118).

Increased proinflammatory cytokines such as IL-1, IL-2, IL-6, IL-7, IL-10, are frequently observed in many COVID-19 patients (119), which is closely related to the acute respiratory distress syndrome (ARDS), multiorgan failure and other severe symptoms. Recombinant IL-1 receptor antagonist (rIL-1Ra, Anakinra) is used to dampen IL-1 induction in severely ill patients (120). Other potential therapeutic targets (121) are IFN-γ (emapalumab, anti-IFN-γ monoclonal antibody) and granulocyte–macrophage colony-stimulating factor (GM-CSF) [TJ003234 and gimsilumab, Anti-GM-CSF monoclonal antibodies (122)], IL-6 [siltuximab, chimeric monoclonal antibody (123)]. Colchicine may also be appropriate in the treatment of COVID-19, as it can inhibit neutrophils, IL-1β and the inflammation/thrombosis interface (124). The application of these anti-inflammatory therapies is helpful in preventing severe outcome.

Other immunotherapies, such as convalescent plasma therapy (125), intravenous immunoglobulin (IVIG) therapy (126), mesenchymal stem cells (MSCs) therapy, are also under investigation for the treatment of COVID-19 (127). Moreover, the efficacy of these therapies against Omicron has not yet been proven and more clinical results are required to draw conclusions. Future immunotherapy may also focus on the ACE2-targeting antibody which has been reported to suppress Omicron (128).

## Virus immune evasion strategies

Insight into viral immune evasion is critical to understanding the pathogenesis of the virus and the challenges facing the immune system and vaccines. SARS-CoV-2 has evolved countermeasures against innate defenses and employed a combination of evasion strategies. Emerging evidence suggests that SARS-CoV-2 infection leads to dysregulation of several types of IFNs which may enhance viral infection. ORF9b downregulates the type I IFN response by inhibiting the IκB kinase alpha (IKKα)/β/γ-NF-κB signaling pathway (129). Age-related IFN dysregulation is also observed in COVID-19 patients, which may explain the susceptibility of elderly patients to SARS-CoV-2 (130). The more proportion of M1-like alveolar macrophages (AMs) may facilitate viral spread and pro-inflammatory responses (131–133). Besides, AMs are even incapable of detecting SARS-CoV-2 and producing IFN response compared to Influenza A virus and Sendai virus (134). SARS-CoV-2 also targets pathways for NK cell receptors, and the signaling of their ligands, apoptosis to escape NK cell-mediated clearance (135). However, there is still controversy about the expression of NK cells in the severe patients. NK cells are found to be decreased in COVID-19 patients (136, 137), while another study detects adaptive NK phenotypes in patients with severe disease (138). In COVID-19 infection, increasing neutrophil numbers and recruitment to lungs are commonly considered to be related to the severity and poor prognosis (139). However, the research on how neutrophils respond to the SARS-CoV-2 is relatively scarce. Different VOCs may differ in the activation intensify of neutrophils, which may be explained by the various severity degree of VOCs (140). Moreover, ORF8 can bind monocytes to decrease the capacity of antibody-dependent cellular cytotoxicity (ADCC) (141). These evidences imply that the aberrant activation and concentration of neutrophils may dysfunction immune system and promote the virus immune escape.

T and B cells normally target viral antigens via the major histocompatibility complex (MHC) on antigen presenting cells (APCs), thereby activating the adaptive immunity (142). However, this immunity is somewhat fragile, as even a single mutation in the epitopes of CD8^+^ and CD4^+^T cells is sufficient to induce cellular immune escape (54, 143). SARS-CoV-2 has developed several strategies to impair T cell participation: (1) accelerating disruption or downregulation of MHC-1 via ORF8 mutant proteins (144); (2) weakening cytotoxic T lymphocyte (CTL) response via mutations of the CD8+ T-cell epitope (145, 146). (3) destructing T-cells and lymphoid organs and causing lymphopenia (147). Mutated epitopes may no longer be recognized by pre-existing CD8^+^ T cell immunity, as mutations in SARS-CoV-2 mainly impaired CD8^+^ T cell recognition (148). As for B cells, mutations in the spike protein can significantly affect the efficacy of antibodies, but studies on how the virus escapes B cell antibodies need to be further explored.

## Cytokine storm syndrome

Some COVID-19 patients experience sudden and rapid deterioration due to cytokine storm syndrome (CSS). CSS includes lude familial/primary and secondary hemophagocytic lymphohistiocytosis (HLH), macrophage activation syndrome (MAS), infection-associated hemophagocytic syndrome, cytokine release syndrome (CRS), and cytokine storm (CS) (149). Innate and adaptive immune cells are involved in the genesis of CSS and IFN-γ, IL-1, IL-6, TNFα, and IL-18 are considered to be the major elevated cytokines (150). HLH is a potentially life-threatening disorder characterized by uncontrolled activation of cytotoxic T cells, NK cells and macrophages (151). The pathogenesis of MAS is attributed to elevated pro-inflammatory cytokines, most particularly IL-6, IL-1β and IL-18 (152). Over-activated macrophages induce pro-inflammatory cytokines, such as TNF α, IL-1, IL-6, and IL-18, and trigger the cytokine storm. Lung macrophages in severe COVID-19 patients may cause local inflammation by recruiting monocytic cells and neutrophils (153). Subsequently, the macrophage activation leads to the expansion and activation of T cells, particularly CD8^+^ cytotoxic T cells, which in turn promote further macrophages activation (154). Excessive IL-6 can downregulate NK cells, which lowers the level of perforin, impairs the immunomodulatory effect of CD8^+^ T cell IFN-γ expression and the ability to eliminate viral triggers (155, 156). Neutrophils produce neutrophil extracellular traps, which facilitates the cytokine storm (150). Mast cells, neurons, glial cells, and endothelial cells are also involved in the induction of inflammatory cytokines (157). Lymphopenia is related to reduced total T cells, CD4^+^ T cells, CD8^+^ T cells, NK cells, and elevated Th17 cells (158, 159). Increased IL-6 can promote the differentiation of Th17 cells and amplify cytokine storm during viral infection (160).Th1 cells also participate in the cytokine storm by producing large amount of IFN-γ (161). It is noteworthy that multisystem inflammatory syndrome in children (MIS-C) is a unique challenge of this pandemic (162), characterized by overwhelming systemic inflammation, fever, hypotension, cardiac dysfunction and neurological complications (163). The incidence of MIS-C in Omicron is less frequent (162), possibly due to enhanced host immunity after COVID-19 vaccination (162). However, the cause of this postinfectious syndrome remains unclear. This possible mechanism is due to a poor antibody response upon first exposure to SARS-CoV-2 (164) and circulating low levels of SARS-CoV-2 replication (165).

## Asymptomatic infection

About 7.9% -61.0% persons remain asymptomatic when receiving positive PCR tests (166), possibly due to the evolution of SARS-CoV-2 (167) and increased coverage of vaccines (168). A single cell RNA sequencing (scRNA-seq) has revealed the enhanced epithelium barrier function, mild inflammation and local CD8^+^ T cell response in asymptomatic carriers, which may also be the reason for the clearance of SARS-CoV-2 without causing disease (169). Children and females are found to be more likely to be asymptomatic and act as unknown carriers (170). Usually, early development of a cytotoxic CD8^+^ T cell response is associated with milder disease (171) and early moderate type I IFN response may control SARS-CoV-2 earlier (172). Therefore, a stronger initial immune response may be positively related to asymptomatic status. It is noteworthy that asymptomatic individuals are not characterized by weak antiviral immunity; on the contrary, they have similar frequencies of SARS-CoV-2-specific T cells as symptomatic individuals (173). SARS-CoV-2-specific T cell responses also generate a higher level of IFN-γ and IL-2, implying the important role of IFN-γ in the early stage of antiviral infection (173). Asymptomatic patients with increased levels of XCL1, XCL2 and IFN-γ have a significant increase in CD56^bri^CD16^-^ NK cells than do moderate and severe subjects. In contrast, the SARS-CoV-2 RBD-specific memory B response in asymptomatic patients is less frequent than that in symptomatic individuals (174). In some cases, the viral load of asymptomatic persons is similar to that of symptomatic persons, implying similar viral transmission ability (175, 176).

## Main predisposing factors of the host

Host factors, including age, gender, genetics, and comorbidities, play a significant role in susceptibility to viral infection and disease pathogenesis. Understanding factors that make host susceptible to SARS-CoV-2 can provide new ideas for its pathogenesis and precision treatment. As fewer studies focus on the effect of susceptible factors on COVID-19, we select factors with more literature for discussion.

### Age and sex

The effect of age and susceptibility to COVID-19 is still insufficient. The relationship between age and ACE2 expression is controversial in several studies (177–179). Age-related immunosenescence is thought to be the main cause of increased susceptibility to infection, such as age-related decline of *de novo* T cell responsiveness (180–182). Infant and young children usually have milder clinical courses, but more vulnerable to bacterial and viral infections (183). Other elements, such as age-related physiological and anatomical changes in the respiratory tract with the manifestation of ciliary dysfunction and weakened respiratory muscle strength, may also make the elderly more susceptible to SARS-CoV-2 infection (178).

Recent studies have reported that men are more affected by COVID-19 than are women (184). Outbreaks of SARS and MERS have also shown a male predominance in disease susceptibility (185). Male mice were also found to be more susceptible to SARS-CoV-2 infection without the confounding effects of smoking (186). Sex differences occur in immune response due to distinct genes and hormones (187). Testosterone in men has been shown to inhibit the expression of pro-inflammatory factors, including IL-1-β, IL-6, C-reactive protein (CRP), and TNF-α (188–192), which delays an effective immune response. The SRY gene may increase male susceptibility to COVID-19 (193). The upregulation of TMPRSS2 by androgens may also explain the increased susceptibility of males to COVID-19 (194). On the other hand, several studies have supported the immunologic protective effects of estrogen in females (195–200), which may be explain why females are less susceptible to SARS-CoV-2 infection. There are several pathways by which estrogen may impact the immune response: (1) induce pulmonary vasodilation by attenuating the vasoconstrictor response to various stimuli, such as hypoxia (200). (2) lower ACE2 and TMPRSS2 levels to alleviate infection (201). Overall, the sex differences in the susceptibility to COVID-19 need larger cohorts to reduce sample selection bias.

### Micronutrients

Moderate vitamins and minerals usually play a protective factor in biochemical processes and have anti-inflammatory, antioxidant, antiviral and antibacterial activities, while malnutrition and undernourishment can impair immunity and increase susceptibility to infection (**Table 1**).

**Table 1:** Role of micronutrients in COVID-19 susceptibility.

Nutrient	Function against COVID-19	Deficiency effect on COVID-19 susceptibility	References
Vitamin A	Maintains the structural barrier of mucosal cells in the skin, respiratory tract, and digestive tract Maintains the optimal function of immune cells in innate and adaptive immunity	Increases susceptibility	(202, 203)
Vitamin C	Stimulates oxygen free radical scavenging activity in the skin Enhances epithelial barrier function Inhibits the expression of ACE2 Limits the entry of SARS-CoV-2 in human small alveolar epithelial cells	Increases susceptibility	(204, 205)
Vitamin D	Inhibits SARS-CoV-2 replication in the host Affects the synthesis of cytokines Lowers ACE2 receptor expression	Increases susceptibility	(206, 207)
Vitamin B12	Inhibits NSP12 polymerase activity of SARS-CoV-2	Increases susceptibility	(208)
Zn	Inhibits the enzyme RNA polymerase Maintains the integrity of the immune barrier Improves the cytotoxic activity of NK cells	Increases susceptibility, particularly in children and the elderly	(209, 210)
Fe	Participates in the activation of immune cells	Increases susceptibility	(211, 212)

Vitamin A may help clear SARS-CoV-2 by maintaining the optimal immune functions and prevent lung infection by maintaining the integrity of the mucosal barrier (202, 203). Vitamin C may protect humans from SARS-CoV-2 by limiting viral entry into human small alveolar epithelial cells, stimulating oxygen free radical scavenging activity in the skin, and improving epithelial barrier function (204, 205). Vitamin B12 can suppress the NSP12 polymerase activity of SARS-CoV-2 (208). Vitamin D can limit SARS-CoV-2 replication and prevent respiratory viral infections, by down-regulating the ACE2 receptor, reducing cytokine storm symptoms and lung inflammatory response (206, 207). Zinc supplementation has been reported to reduce infection, while its deficiency lead to humoral and cell-mediated immune dysfunction and increase susceptibility (209, 213). Iron is an important component of enzymes involved in immune cell activation, and its deficiency cause increased susceptibility to infection, especially with intracellular pathogens (211, 212).

### Comorbidities and associated diseases

Obesity, a global epidemic, is positively associated with SARS-CoV-2 infection (214). Virions may interact with the excessive renin-angiotensin-aldosterone system (RAAS) and insulin resistance (IR) in obese patients, leading to increased infection via ACE2 (215). The elevated adipose tissue in obese people also results in increased ACE2 expression, facilitating viral entry and spread to host cells (216). Other studies have suggested that obesity may delay the immune response and increase the likelihood of the infection (217).

As a leading chronic disease (218), diabetic patients are more likely to be infected with SARS-CoV-2 (219, 220). ACE2 is highly expressed in patients with diabetes, especially those taking either ACE inhibitors or angiotensin II type-1 receptor blockers (ARBs) (221). Studies have shown that the diabetes status impairs the chemotactic function of neutrophils and causes the respiratory microangiopathy to increase susceptibility to lower respiratory tract infections (222, 223). Elevated plasmin levels are also a common feature in patients with diabetes, which may enhance the infectivity of SARS-CoV-2 by accelerating the entry, fusion, replication, and release of SARS-CoV-2 in respiratory cells (224, 225). Diabetes also impairs the immune system by premature recruitment of neutrophils and macrophages, reduced NK cell activity, Th1 cell-mediated immune activation delay and hyperinflammatory response, leading to delayed virus clearance (226–232).

Patients with asthma are susceptible to SARS-CoV-2, resulting in severe asthma exacerbation (233, 234). One possible reason is the depressed type-II IFN immune activity, characterized by reduced interferon synthesis (235–237). In contrast, the expression of ACE2 and TMPRSS2 shows no difference between asthmatic patients and healthy donors, which may not be identified as an influencing factor (238). More researchers are focusing on the outcome of patients with chronic obstructive pulmonary disease (COPD). SARS-CoV-2 infection may also exacerbate COPD, which is characterized by severely reduced ciliary function and worsening symptoms (239). Interestingly, smokers with COPD patients had higher ACE2 levels (240). However, smoking may be a confounding factor in this study, so further studies are needed.

Patients with systemic autoimmune diseases are susceptible to severe infection with COVID-19 (241), mainly due to attenuated adaptive and innate immune responses and continuous use of immunomodulatory drugs. Patients with multiple sclerosis are twice more likely to be infected with SARS-CoV-2 and require hospitalization as those without the disease, which may be due to the associated etiology and drug therapy management (241–243). Treatment with these immunomodulatory drugs, including IFN-β, glatiramer acetate, and teriflunomide, can improve immunosuppression and reduce predisposition to viral infection, while the use of dimethyl should be used with caution as it may cause lymphopenia, and increase the potential threat (244).

## Summary and outlook

We have attempted to discuss the core issues of innate and adaptive immune responses to the novel SARS-CoV-2. The current mechanisms of PRRs, including TLRs, RLRs and NLRs in viral recognition and effective innate immunity mediation against COVID-19 and their immunopathology towards COVID-19 are comprehensively concluded in this paper. Further mechanistic insights into the associations between the magnitude of clinical manifestations and innate immunity are a high priority. Meanwhile, T and B cells are elicited by SARS-CoV-2 antigens to eliminate the virus and generate protective antibodies. The majority of studies have shown that the limited level of antibody titers after a long period. The T and B cell immune events and their span of durable immunity following vaccination are unified in different findings and therefore studies of SARS-CoV-2-specific CD4^+^T cells, CD8^+^ T cells and antibodies together in larger cohort of patients at different disease phases are needed to be further carried out. Age differences concerning adaptive immunity in COVID-19 also need to be further investigated. Both cross-reactivity of antibody and T cell immune responses have been demonstrated in many studies, and the further research about cross-reactivity of children against COVID-19 may support pediatric vaccination regimens. We then highlight the possible mechanisms by which SARS-CoV-2 evades the immunity to gain a deeper understanding of the viral pathogenesis and to discover early therapeutic interventions. We also summarize the clinical symptoms of cytokine storm syndrome and the possible mechanisms of asymptomatic infection. Much more research is needed to understand the long-term effects of COVID-19 in children. What is more, we review the current understanding of the major host predisposing factors to COVID-19 in the limited articles. The factors, including sex, age, micronutrients, and comorbidities may make the host more susceptible to the virus and these complicated interactions further expand the understanding of the disease pathogenesis. Identification of these factors is crucial to classify susceptible populations and carry out interventions.

## Author contributions

JS, JF, YZ and GC conceived the work, wrote the original manuscript, revised, and finalized the manuscript. DJ and ZN reviewed and edited the manuscript. ZZ supervised and reviewed the original manuscript. All authors have read and agreed to the published version of the manuscript. All authors contributed to the article and approved the submitted version.
